# A Genome-Wide Association Study Provides New Evidence That *CACNA1C* Gene is Associated With Diabetic Cataract

**DOI:** 10.1167/iovs.16-19332

**Published:** 2016-04-28

**Authors:** Cheng Chang, Kaida Zhang, Abirami Veluchamy, Harry L. Hébert, Helen C. Looker, Helen M. Colhoun, Colin N. A. Palmer, Weihua Meng

**Affiliations:** 1 Division of Population Health Sciences Ninewells Hospital and School of Medicine, University of Dundee, Dundee, United Kingdom; 2 Centre for Pharmacogenetics and Pharmacogenomics, Ninewells Hospital and School of Medicine, University of Dundee, Dundee, United Kingdom

**Keywords:** genome-wide association study, cataract, diabetes, genetics

## Abstract

**Purpose:**

Diabetic cataract is one of the major eye complications of diabetes. It was reported that cataract occurs two to five times more frequently in patients with diabetes compared with those with no diabetes. The purpose of this study was to identify genetic contributors of diabetic cataract based on a genome-wide association approach using a well-defined Scottish diabetic cohort.

**Methods:**

We adapted linked e-health records to define diabetic cataract. A diabetic cataract case in this study was defined as a type 2 diabetic patient who has ever been recorded in the linked e-health records to have cataracts in both eyes or who had previous cataract extraction surgeries in at least one eye. A control in this study was defined as a type 2 diabetic individual who has never been diagnosed as cataract in the linked e-health records and had no history of cataract surgeries. A standard genome-wide association approach was applied.

**Results:**

Overall, we have 2341 diabetic cataract cases and 2878 controls in the genetics of diabetes audit and research in Tayside Scotland (GoDARTS) dataset. We found that the *P* value of rs2283290 in the *CACNA1C* gene was 8.81 × 10^−10^, which has reached genome-wide significance. We also identified that the blood calcium level was statistically different between diabetic cataract cases and controls.

**Conclusions:**

We identified supporting evidence that *CACNA1C* gene is associated with diabetic cataract. The role of calcium in the cataractogenesis needs to be reevaluated in future studies.

Cataract is defined as a loss of normal transparency of the crystalline lens in the eye due to opacification or optical dysfunction.^1^ It affects light transmission and results in a deteriorated vision.^1^ According to the visual impairment data reported by the World Health Organization in 2012, cataract is the leading cause of global blindness, accounting for 51% of the overall cases, far higher than blindness caused by glaucoma (8%) and by age-related macular degeneration (5%).^2^ In addition to being a major ocular disorder, cataract also puts heavy economic burdens on health care systems. For example, the total direct medical cost of cataract was $6.8 billion in 2004 in the USA, representing 42% of the total direct medical cost of all visual disorders in that year.^3^ Cataract is also associated with many ophthalmic and general health conditions such as myopia and a high rate of falling.^4,5^

Diabetic cataract is one of the major eye complications of diabetes. It was reported that cataract occurs two to five times more frequently in patients with diabetes compared with nondiabetic populations.^6,7^ Around one in four of the late onset diabetic patients will undergo a cataract surgery in a 10-year interval.^7^ Multiple epidemiological studies have suggested risk factors for diabetic cataract such as age, sex, body mass index (BMI), duration of diabetes, educational level, smoking history, macroalbuminuria, HbA1c, and hemoglobin.^8,9^ Identifying potential risk factors of diabetic cataract can help in the generation of therapeutic interventions and supplementary treatment methods and ease the disease burden from an economic and public health perspective.^10^ The genetic studies of cataract have been mainly focused on congenital cataract or cataract caused by a single mutation and around 30 loci have been identified so far.^11^ However, these loci or genes are less likely to be relevant to diabetic cataract since diabetic cataract is normally considered as a complex disorder, affected by both genetic and environmental factors like other diabetic complications such as diabetic neuropathic pain.^12,13^ Lin et al.^14^ proposed 15 loci associated with diabetic cataract across the genome in the Chinese population through a small scale genome-wide association study (GWAS). These loci contain the candidate genes *PPARD*, *CCDC102A*, *GBA3*, *NEDD9*, *GABRR1/2*, *RPS6KA2*, *HSPA8P8*, *TAC1*, *GALNTL1*, and *KIAA1671*, which are either involved with the mechanisms of regulating blood sugar or the formation of cataract.^14^ Understanding the genetic factors associated with diabetic cataract would help to identify the underlying mechanisms and potentially identify molecular targets for pharmacological research.

The purpose of this study was to identify genetic contributors of diabetic cataract based on a genome-wide association approach using a well-defined Scottish diabetic cohort.

## Methods

### Participants

The genetics of diabetes audit and research in Tayside Scotland (GoDARTS) project was originally created to identify genetic factors for diabetes and its complications. All participants complete a lifestyle questionnaire, undergo a baseline clinical examination, and provide their biological samples (blood and urine). In addition, they allow researchers to use their health information and their biological samples for research purposes and give permissions to link their personal information anonymously to their national health service (NHS) medical records. The medical records include their prescribing history, general practice clinic visits, hospital admissions, and outpatient appointments. Furthermore, their personal information is anonymously linked with the Scottish Care Information-Diabetes Collaboration (SCI-DC) database, which is an electronic health record system designed to keep track of local diabetic patients and help health professionals to provide better care in Scotland. Written informed consent was obtained from all participants. Further information about the GoDARTS project is available in the public domain at http://diabetesgenetics.dundee.ac.uk/. The research followed the tenets of the Declaration of Helsinki. The ethics approval has been granted by Tayside Committee on Medical Research Ethics (REC reference 053/04).

The GoDARTS project has recruited 9439 diabetic patients so far, among which 6927 were already genotyped by DNA chips. All GoDARTS participants' health information was anonymously linked with their NHS and SCI-DC medical records from their enrollment until June 2011.

### Definitions of Diabetic Cataract Cases and Controls

A diabetic cataract case in this study was defined as a type 2 diabetic patient who had ever been recorded in the linked e-health records as having cataracts in both eyes or who had previous cataract extraction surgeries in at least one eye. In the linked e-health records, there is no indication of the subtypes of cataract such as cortical cataract, nuclear cataract, and posterior subcapsular cataract as well as the severity of cataract. The diagnosis of cataract is mainly made by clinicians in the annual national retinal screen service.

A control in this study was defined as a type 2 diabetic individual who has never been diagnosed as cataract in the linked e-health records and had no history of cataract surgeries.

No other criteria (such as trauma and infection) were considered when defining cases and controls.

### Genotyping and Quality Control

The project GoDARTS adapted two types of DNA chips to genotype its diabetic individuals. The Affymetrix SNP 6.0 chips (used on 3673 subjects; Affymetrix, Santa Clara, CA, USA) were funded by the Wellcome Trust Case Control Consortium 2 (WTCCC2) project^15^; the Illumina chips (used on 3254 subjects; OmniExpress BeadChip kit; Illumina, Inc., San Diego, CA, USA) were funded by the surrogate markers for micro- and macrovascular hard endpoints for innovative diabetes tools (SUMMIT) project.^16^ Genotype data quality controls were based on the standard protocols that were established for the WTCCC2 studies^15^ and the SUMMIT studies.^16^

### Statistical Analysis

Software SHAPEIT and IMPUTE2 were used to impute nondirectly genotyped single nucleotide polymorphisms (SNP) with reference files from the 1000 genomes phase I datasets (both directly genotyped SNPs and reference files were based on genome assembly National Center for Biotechnology Information b37).^17,18^ To filter out poorly imputed SNPs, a *r*^2^ < 0.3 is applied as it is the lower threshold value recommended by IMPUTE2 and we wanted to maximize the number of SNPs for further analysis.

The primary data manipulation software was PLINK and routine quality control steps were frequently applied during data analyses (e.g., removing SNPs with less than 95% genotyping call rate, SNPs with minor allele frequency less than 1%, SNPs that failed Hardy–Weinberg tests *P* < 0.000001 [based on control samples only], and removing individuals with more than 5% genotype data missing).^19^ Single nucleotide polymorphisms on sex chromosomes and mitochondrion were excluded. Multidimensional scaling analysis integrated in PLINK was used to detect population stratification. A lambda value was calculated to indicate the level of stratification. The lambda value should be very close to 1 indicating a minimum ancestry mixture. Samples with pi-hat > 0.125 were discarded due to relatedness. A logistic regression test with multiple covariates was applied to generate *P* values for SNP associations. A value of *P* < 5 × 10^−8^ is considered to be significant.

Other GWAS-related software used in our study were: SNPnexus for SNP functional annotation,^20^ HaploView for generating Manhattan plots and linkage disequilibrium (LD) blocks,^21^ and SNPEVG for generating corresponding quantile-quantile (q-q) plot to evaluate differences between cases and controls caused by potential confounders (different genotyping laboratories, different DNA extraction methods, etc.).^22^ Means of age, BMI, cholesterol, triglycerides, high-density lipoprotein (HDL), low-density lipoprotein (LDL), and HbA1c were compared between cases and controls using independent *t*-test (SPSS 22; IBM Corp., Armonk, NY, USA). Sex difference was compared using a χ^2^ test. Blood calcium level was compared later using an independent *t*-test. The whole workflow was shown in Supplementary Figure S1.

## Results

In this general diabetic cohort, we identified 2501 type 2 diabetic patients with cataract and 3032 controls with no cataract according to the linked e-health records after removing type 1 diabetic patients and patients with no genetic data. Then, after filtering out related samples and outlier samples detected by population stratification analyses, we had 2341 diabetic cataract cases (males = 1249, females = 1092) and 2878 controls (males = 1655, females = 1223). The prevalence of diabetic cataract in our cohort was 44.9%. We compared the means of sex, age, BMI, cholesterol, triglycerides, HDL, LDL, HbA1c between cases and controls. There were statistical differences in sex, age, BMI, HDL, LDL, HbA1c between cases and controls while there was no statistical difference in cholesterol and triglycerides (Table 1).

**Table 1:**
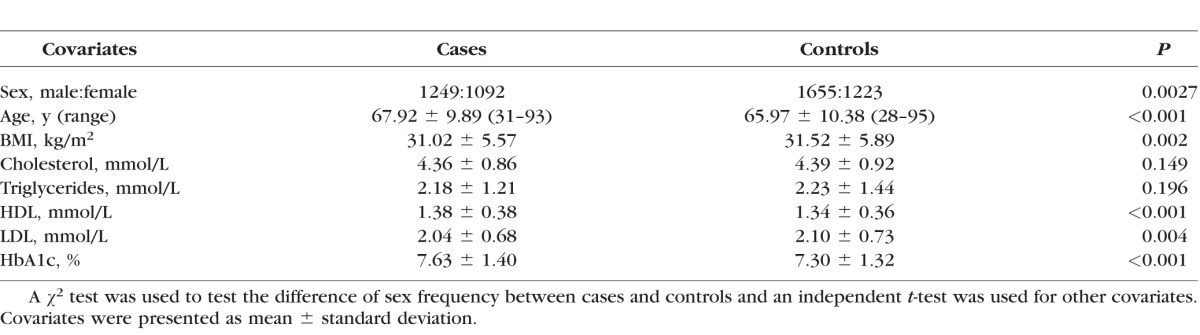
Clinical Characteristic of Diabetic Cataract Cases and Controls

Affymetrix SNP6.0 chips contain 704,847 directly genotyped and quality-controlled SNPs and Illumina OmniExpress chips contain 601,394 directly genotyped and quality-controlled SNPs. Altogether, 6,398,685 genotyped and imputed SNPs were available for further analysis, after routine quality control of genotyping and imputation. The lambda value, which indicates the level of population stratification, was 1.06 and therefore no further adjustments for population stratification was needed. We then performed logistic regression tests on all SNPs, with age, sex, BMI, HDL, LDL, and HbA1c as covariates. We identified rs2283290 in the *CACNA1C* gene with a *P* value of 8.81 × 10^−10^ and an odds ratio (OR) of 0.72 (A allele, 95% confidence interval: 0.66–0.80; Fig.). We calculated the correlation between rs2283290 and 10 upstream and 10 downstream SNPs using PLINK and found that it was not in LD (*R*^2^ > 0.8) with any of its nearby SNPs (Supplementary Figure S2). We also downloaded the linkage information of this SNP from HapMap and found the SNP was not in LD with its nearby SNPs as well (Supplementary Figure S3). The plot q-q of the association results was shown in the Supplementary Figure S4.

**Figure:**
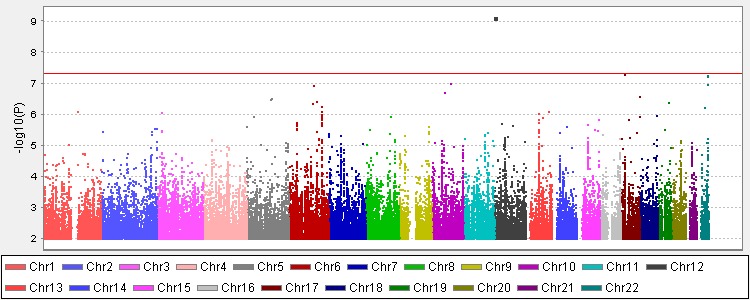
The Manhattan plot of the GWAS on diabetic cataract (2341 cases and 2878 controls) SNPs with *P* < 0.01 were not included. The *red line* represents a *P* value of 5 × 10^−8^ in the plot.

We extracted the baseline blood calcium values of cases and controls from the linked e-health records (only 4222 individuals have those values recorded). We found that there was a statistical difference of the blood calcium levels between diabetic cataract cases and controls in both men and women (*P* = 0.001; Table 2).

**Table 2:**
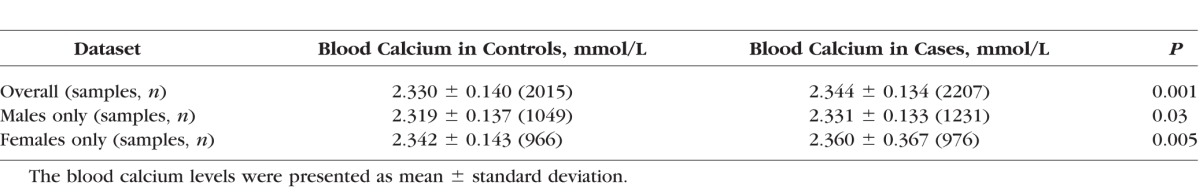
Comparison of Blood Calcium Levels Between Diabetic Cataract Cases and Controls

## Discussion

We performed a GWAS on diabetic cataract using a Scottish diabetic cohort based on phenotype information from linked e-health records and genetic information from DNA chips. We found that *CACNA1C* gene may be involved with diabetic cataract.

All diabetic patients in Scotland are invited to have retinal screening annually. During the screening, clinicians determine whether patients have cataracts or not, along with the diagnosis of diabetic retinopathy. However, in the case of a diagnosis of a cataract, the specific subtype of the cataract or the severity of the cataract is not reported. In fact, cataract appears more often in a mixed format—a combination of nuclear cataract, cortical cataract or posterior subcapsular cataract—than a single entity in clinical settings.^23^ It was reported that around one in three cataracts are a mixed type in a diabetic population.^9^ Therefore, the phenotype used in our study is “any cataract,” including mixed cataracts and any subtypes of cataracts. The prevalence of diabetic cataract in our cohort is 44.9%, which is matched with the prevalence of 47.9% for diabetic cataract in an Indian diabetic population.^9^ In principle, using a specific subtype of cataracts will have a higher ability to identify relative genes for genetic studies while in reality, these advantages normally are offset by reduced sample size and correspondingly reduced study power. In this study, using the CaTS power calculator, we had 80% power based on 2341 cases and 2878 controls, assuming a minor disease allele frequency of 0.15, a genotypic relative risk of 1.21, a prevalence of diabetic cataract in the diabetic population of 0.45, and the significance level of 5 × 10^−8^.^24^

We identified that the smallest *P* value was 8.81 × 10^−10^ with an OR of 0.72 for rs2283290 in the *CACNA1C* gene, which reached GWAS significance (*P* < 5 × 10^−8^). It was a sporadic SNP with no supporting SNPs in the plot. The linkage disequilibrium analysis of the cohort revealed that this SNP had small *R*^2^ values (indicating levels of LD) with its nearby SNPs (10 upstream and 10 downstream SNPs). This finding was matched with the HapMap Caucasian dataset, which also shows that rs2283290 and its nearby SNPs are not in LD, indicating that the significance is not caused by a genotyping error (Supplementary Figure S3). The closest SNP in LD with rs2283290 is rs2239032 (10 kb away, *R*^2^ = 0.97) in the HapMap dataset, but the *R*^2^ score is 0.70 between the two SNPs in our GWAS dataset. This can be due to the population difference since we used a diabetic population while HapMap used a general Caucasian population. The imputation score of this SNP in our dataset is 0.970 (imputed in Affymetrix chips only since the SNP is directly genotyped in Illumina chips), indicating a good imputation result. The *CACNA1C* gene encodes a protein called the alpha-1 subunit of a voltage-dependent, dependent L-type calcium channel (also known as CaV1.2). The calcium channel CaV1.2 is expressed and distributed in the epithelium and cortical fiber cells in the mouse lens, especially in the short arms of the hexagonal lens fibers.^25^ The channel CaV1.2 influences different cellular responses as it relates to the regulation of intracellular calcium.^26^ Inhibition of the L-type calcium channel with felodipine or nifedipine has been reported to induce progressive cortical cataract formation and be associated with decreased lens weight in ex vivo mouse lenses.^25^ In addition, L-type calcium channel inhibitors have been reported to delay formation of diabetic cataract in rodents.^27^ Proliferation of human lens epithelial cells is also inhibited by blocking of calcium channels.^28^ Researchers have confirmed there was a more than a 23-fold increase in total lens calcium with cataract.^29^ Calcium homeostasis has been confirmed to be impaired in lens from diabetic rats and diabetic rabbits.^30,31^ In this study, we could not measure calcium in the lens, but we found that the total blood calcium was statistically different between diabetic cataract cases and controls both in males and in females. The result represented statistical significance although it requires further evidence to discover if there is a physiological reason behind it. Genetic variants in the *CACNA1C* gene have been reported in multiple diseases such as arrhythmia, bipolar disorder, and schizophrenia.^32–34^ Further studies are needed to explore the functional role of *CACNA1C* in diabetic cataract.

At the moment, the mechanism of diabetic cataract is not clear. Some evidence has supported the polyol pathway mechanism, which produces overloaded sorbitol from glucose first and then the overloaded sorbitol increases the osmotic stress in the lens fiber leading to its swelling and rupture.^35,36^ The pathway was widely indicated for the microvascular damage of the diabetic complications such as diabetic retinopathy.^37^ However, the result of a large clinical trial on this pathway is negative for diabetic retinopathy.^38^ This indicates the involvement of other possible mechanisms such as pathways including calcium. As we identified in this study, the blood calcium level is statistically different between diabetic cataract cases and controls in men and women. Combining the fact that the calcium level is significantly elevated in the lens during cataract, calcium might have a greater role in the cataractogenesis than we expected.

It is also very challenging to differentiate age-related cataract from diabetic cataract since a type 2 diabetic population is normally an elder population as well. The development of cataract in a diabetic patient could be partly due to the aging process. At the moment, we do not know the interaction between the aging process and diabetic contribution in terms of cataractogenesis. Do they work independently, or do they accelerate each other's role in cataractogenesis? Therefore, a wiser phenotyping strategy should be established to identify diabetic cataract in an elder diabetic population.^39^

We also extracted our results of the SNPs which were reported by other GWAS on cataract such as Lin et al.^40^ (diabetic cataract), Liao et al.^11^ (age-related cataract), and Ritchie et al.^41^ (age-related cataract; Supplementary Table S1). The statistical *P* values of these SNPs in our study were not small enough to merit further investigation.

In conclusion, we identified that *CACNA1C* gene is associated with diabetic cataract in a Scottish diabetic cohort using a GWAS approach. We also reported that the blood calcium level is significantly different between diabetic cataract cases and controls indicating the potential role of calcium in the cataractogenesis.

## Supplementary Material

Supplement 1

Supplement 2

Supplement 3

Supplement 4

Supplement 5
