# Supplementary figures and images for: A Genome-Wide Association Study Provides New Evidence That CACNA1C Gene is Associated With Diabetic Cataract
Source: Invest Ophthalmol Vis Sci. 2016 Apr 28;57(4):2246–50. doi: 10.1167/iovs.16-19332 (PMC4855826; doi:10.1167/iovs.16-19332)

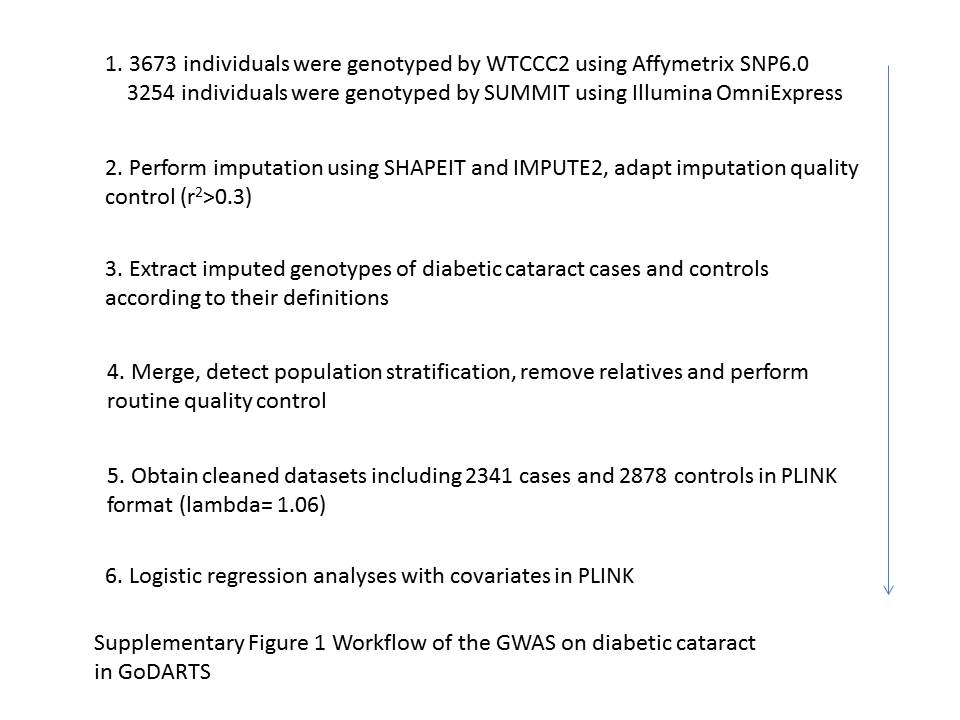

Supplement: Supplement 1 [file i1552-5783-57-4-2246-s01.jpg]

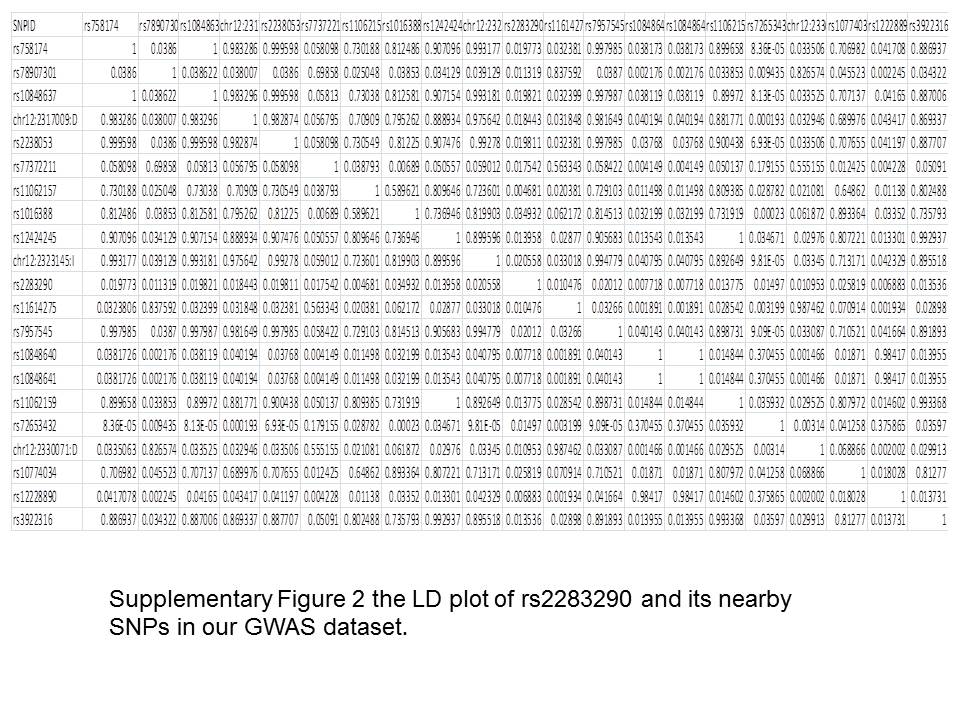

Supplement: Supplement 2 [file i1552-5783-57-4-2246-s02.jpg]

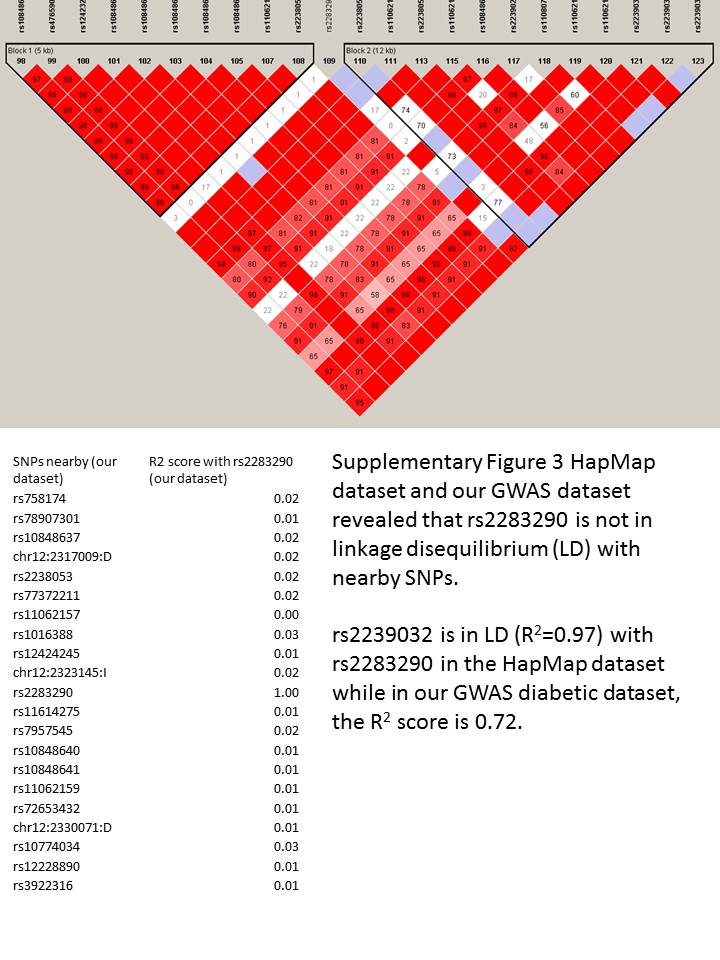

Supplement: Supplement 3 [file i1552-5783-57-4-2246-s03.jpg]

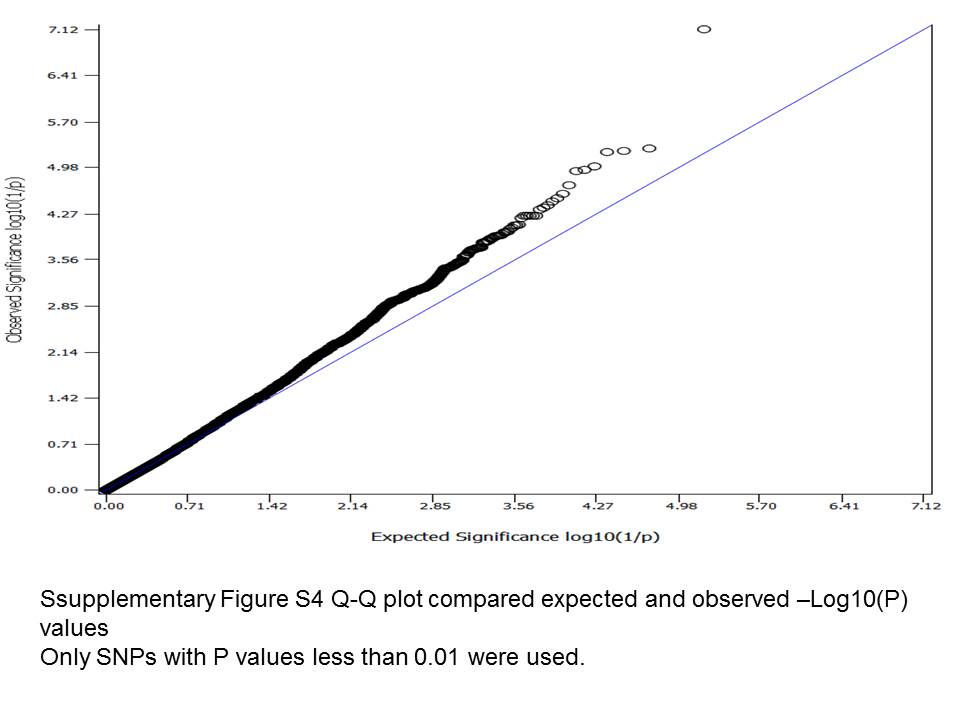

Supplement: Supplement 4 [file i1552-5783-57-4-2246-s04.jpg]
