# Supplementary material for: A Genome-Wide Association Study Provides New Evidence That CACNA1C Gene is Associated With Diabetic Cataract
Source: Invest Ophthalmol Vis Sci. 2016 Apr 28;57(4):2246–50. doi: 10.1167/iovs.16-19332 (PMC4855826; doi:10.1167/iovs.16-19332)
Supplement: Supplement 5 [file i1552-5783-57-4-2246-s05.pdf]

Chromosome	SNP*	Position	OR*	SE*	L95*	U95*	*P*
1	rs16853148	14242772	0.8238	0.08581	0.6963	0.9747	0.02392
1	rs207145	55808143	1.131	0.06318	0.9994	1.28	0.05121
1	rs991007	62517947	1.027	0.06992	0.8955	1.178	0.7028
1	rs4951508	210829477	1.029	0.04999	0.9334	1.135	0.5609
1	rs10746432	210837691	0.9931	0.04039	0.9175	1.075	0.8646
1	rs1416156	218397580	0.9766	0.04062	0.9018	1.058	0.5595
1	rs6663771	218414043	0.9819	0.04043	0.9071	1.063	0.6508
2	rs4560089	35700888	1.015	0.04376	0.9312	1.105	0.7405
2	rs10195113	40832632	0.9079	0.0859	0.7672	1.074	0.2605
2	rs12612521	124291308	1.003	0.05	0.9096	1.107	0.9488
2	rs16857804	171012115	0.9572	0.04504	0.8763	1.045	0.331
3	rs17008958	71755488	0.8795	0.06016	0.7817	0.9895	0.03279
3	rs1447899	71757417	0.932	0.04551	0.8524	1.019	0.1215
3	rs13074058	184867552	1.031	0.06732	0.9033	1.176	0.6528
4	rs2406040	137590890	1.011	0.04402	0.9274	1.102	0.8046
4	rs2897305	137600017	1	0.04462	0.9163	1.091	0.9989
5	rs160044	88114476	0.992	0.04277	0.9122	1.079	0.851
5	rs3850653	88186954	1.034	0.04769	0.9421	1.136	0.4782
5	rs2277939	153830890	0.9926	0.04265	0.9129	1.079	0.8609
5	rs1472606	175018135	0.9208	0.04289	0.8466	1.002	0.05447
6	rs9379053	6664030	0.9068	0.06146	0.8039	1.023	0.1114
9	rs12347205	92909003	1.006	0.04112	0.9277	1.09	0.8931
9	rs882809	104229795	1.038	0.0426	0.9545	1.128	0.3854
10	rs9299674	32435853	1.022	0.04347	0.939	1.113	0.6093
10	rs4301693	32438199	1.002	0.05826	0.8937	1.123	0.9748
10	rs4388822	86646491	0.8841	0.09046	0.7405	1.056	0.1732
12	rs10778791	81516527	0.9673	0.1243	0.7582	1.234	0.7892
12	rs2574730	81530779	0.9724	0.1244	0.762	1.241	0.8222
12	rs769056	81537653	0.9724	0.1244	0.762	1.241	0.8222
12	rs12099972	94527556	1.061	0.07283	0.9197	1.224	0.418
12	rs2656824	127815086	1	0.04531	0.9152	1.093	0.9958
12	rs2593270	127815178	0.9977	0.04523	0.9131	1.09	0.9599
14	rs1742707	92701716	1.02	0.04031	0.943	1.104	0.6147
15	rs1524876	31263272	1.025	0.03987	0.9477	1.108	0.5406
15	rs1432442	66719269	0.9916	0.07176	0.8615	1.141	0.9059
15	rs4778856	81091879	1.008	0.04055	0.9313	1.092	0.8381
15	rs1993976	98606313	0.9807	0.04107	0.9049	1.063	0.6356
15	rs8027435	98624886	1.039	0.04115	0.9586	1.126	0.3514
15	rs4965818	101832688	1.05	0.04275	0.966	1.142	0.2503
16	rs8044853	81457659	1.007	0.04346	0.9248	1.097	0.8719
16	rs9927153	81470018	1.002	0.04804	0.9116	1.1	0.9736
16	rs933717	87415250	1.054	0.04079	0.9731	1.142	0.1967
17	rs4531770	69653253	0.9193	0.06044	0.8166	1.035	0.1637
18	rs7227421	11749198	1.005	0.1119	0.8071	1.251	0.9649
20	rs1337906	22214821	1.009	0.04293	0.9276	1.098	0.8352
20	rs864184	58326432	0.9606	0.04836	0.8738	1.056	0.4064
20	rs6070943	58355991	0.962	0.05348	0.8663	1.068	0.4693

22	rs926937	27929853	1.11	0.08456	0.9402	1.31	0.2185

Table S1 Our results of the SNPs which were reported by other GWAS on cataract

*SNP: single nucleotide polymorphism; OR: odds ratio; SE: standard error; L95 and U95: lower confidence interval and upper confidence interval of OR.

Among 94 SNPs from Lin et al, Liao et al, Ritchie et al, only 48 SNPs were included in our GWAS dataset. This is due to population difference since Lin et al and Liao et al used non-Caucasian samples.
